# Predictors of operative management in diabetic foot ulcers

**DOI:** 10.1002/jfa2.12024

**Published:** 2024-05-26

**Authors:** Amos Au, Erwin Yii, Ana Andric, Jennifer Wong, Alan Saunder, Ming Yii

**Affiliations:** ^1^ Department of Vascular Surgery Monash Health Clayton Victoria Australia; ^2^ Department of Vascular Surgery Eastern Health Box Hill Victoria Australia; ^3^ Department of Podiatry Monash Health Clayton Victoria Australia; ^4^ Department of Endocrinology Monash Health Clayton Victoria Australia; ^5^ Department of Surgery School of Clinical Sciences at Monash Health Monash University Clayton Victoria Australia

**Keywords:** amputation, CRP, diabetic foot complications, diabetic foot infection/ulcer, osteomyelitis

## Abstract

**Background & Aims:**

Surgery plays a key role in the management of complicated diabetic foot disease (DFD). Currently, indications for medical versus surgical management are poorly defined. Prompt identification of patients who require surgery may reduce morbidities and length of hospital stay. This study aims to analyse factors in DFD that necessitate early surgical interventions.

**Methods:**

All patients admitted under a multi‐disciplinary diabetic foot team in a tertiary institution over 2 years were included in a retrospective case‐control study comparing patients who received medical management and patients who received surgical management. Logistic regression was performed to identify factors associated with surgical management of diabetic foot complications.

**Results:**

Three hundred and forty patients were included. 49% of patients required surgical management. Toe ulceration, elevated C‐reactive protein (CRP), and the presence of osteomyelitis were associated with surgical management. Multivariate analysis calculated an odds ratio (OR) of 1.01 for CRP (*p* < 0.001), OR 2.19 (*p* < 0.019) favouring surgical management for forefoot ulcers, and OR 2.2 (*p* < 0.019) if osteomyelitis was present.

**Conclusions:**

Patients with elevated CRP levels, a forefoot diabetic ulcer and established osteomyelitis were more likely to undergo surgical management. Prompt recognition of these patients has the potential benefit of earlier decision making in definitive surgical interventions.

## 1 INTRODUCTION

Diabetic foot disease (DFD) is a common condition that is increasing in prevalence, which has a major impact on health outcomes for patients [1]. Foot ulceration is a common complication of DFD which has been linked to poor outcomes [2], with studies demonstrating a 40% 5‐year mortality rate following development of an initial foot ulcer in the setting of diabetes [3]. DFD results in approximately 360,000 hospital bed days in Australia each year at the cost of $350 million for inpatient management alone [4].

There is a spectrum of management options for foot ulcers in DFD, from medical management with intravenous antibiotics to surgical amputation. Decision‐making processes may vary between health professionals, units, and internationally, may be dependent on factors such as clinical severity, perfusion status, previous history, and the standard protocols of the treating unit. While it may be clear that surgical management is necessary in severe cases, such as those with systemic inflammatory response syndrome, necrotising fasciitis, deep abscesses, or septic necrosis [5]; the clinical factors that influence decision‐making in non‐urgent cases are not well established. The aim of this study is to analyse the medical and surgical patient cohorts to characterise factors in patients with ulcerative DFD required surgical management, and report any differences between these groups that may have the potential to assist in decision making.

## 2 METHODOLOGY

This is a retrospective case‐control study using data collected from the Monash Health Diabetic Foot Unit (DFU). Monash Health is a tertiary medical centre in south‐east Melbourne and is the primary referral hospital for patients presenting with DFD from this area, as well as from the greater Gippsland region. The DFU is a multidisciplinary service that includes medical specialists such as vascular surgeons, endocrinologists, infectious diseases physicians, consultative orthopaedic and plastic surgery specialists, and allied health specialists such as podiatrists and wound care nurses. This study was approved by the Monash Health ethics committee.

Patients admitted under the DFU over a 2‐year period were identified and added to a database. Further data were collected retrospectively via electronic medical records. Inclusion criteria included all patients with diabetes who had ulcers or tissue loss (such as necrosis or gangrene) of the lower limbs and required inpatient management. Exclusion criteria included patients without diabetes, patients who had ulcers secondary to venous disease, and patients who were under 18 years of age.

Patients were then divided into those who required surgical intervention (cases) and those who did not (controls). Surgical intervention was defined as formal debridement or amputation in the operating theatre. For the purposes of this study, patients that received open or endovascular interventions prior to amputation were included into this group. It should be noted that patients managed in the surgical intervention arm will have received IV and/or oral antibiotics leading up to surgery as per local peri‐operative protocols. Furthermore, patients who received additional antibiotics whilst waiting for surgery were not excluded from this group. Medical management was defined as patients who did not have any open surgical (or endovascular) interventions, and received medical management alone, according to the standard unit protocol. Medical management consisted of IV and oral antibiotics, dressing changes, and minor ward‐based wound debridement(s) as well.

Data collected included demographic details, diabetes status and duration, HbA1c level, comorbidities, C‐reactive protein (CRP) on admission, ulcer characteristics, vascular status, and previous interventions. These are included in Table 1.

**TABLE 1:** Baseline characteristics for all patients included in the study.

Variables	Overall (*n* = 340)
Age (years) (IQR)	65 (29.0, 90.0)
Sex (%)	
Female	94 (28%)
Male	246 (72%)
Body mass index (kg/m^2^) (IQR)	29.6 (19.1, 53.8)
C‐reactive protein (mg/L) (IQR)	32 (0.9, 327.0)
Diabetes status	
Diabetes type (%)	
1	32 (9%)
2	308 (91%)
Duration of diabetes (years) (%)	
<5	34 (12%)
5–10 years	52 (17%)
>10 years	211 (71%)
HbA1c (%) (IQR)	8.1% (5.2, 15.0)
Co‐morbidities	
Hypertension status (%)	256 (76%)
Hypercholesterolaemia (%)	219 (65%)
Peripheral neuropathy (%)	190 (56%)
Peripheral arterial disease (%)	124 (44%)
Ischaemic heart disease (%)	123 (36%)
Cerebrovascular disease (%)	64 (19%)
Current smoker (%)	51 (16%)
Living situation	
Lives alone (%)	78 (23%)
Lives with others (%)	233 (70%)
Nursing home (%)	24 (7%)
Previous vascular diagnoses/interventions	
Below knee arterial disease (%)	131 (41%)
Treated below knee arterial disease (%)	53 (17%)
Above knee arterial disease (%)	96 (30%)
Treated above knee arterial disease (%)	47 (14%)
Treated iliac disease (%)	9 (3%)
Endovascular surgery (%)	75 (23%)
Open vascular surgery (%)	19 (6%)
Ulcer characteristics	
Location (%)	
Foot, or ankle, or leg	133 (41%)
Toes	192 (59%)
Osteomyelitis (%)	101 (30%)
Bilateral foot ulcers (%)	27 (8%)
Vascular status	
One or more foot pulses palpable (%)	312 (97%)
Anterior tibial artery occluded (%)	64 (20%)
Posterior tibial artery occluded (%)	83 (26%)
Peroneal artery occluded (%)	49 (15%)
Iliac disease (%)	16 (8%)

Abbreviation: IQR, interquartile range.

## 3 STATISTICAL ANALYSIS

All statistical analysis was performed using the statistical package Stata IC 15.1 (StataCorp. 2017. Stata Statistical Software: Release 15. StataCorp LLC.). Continuous variables are reported as median, interquartile range (IQR), and minimum and maximum values when the distribution was skewed. Associations between the outcomes and variables was analysed using univariate and multivariate logistic regression. All logistic regression models were adjusted for known confounders including age, gender, diabetes type, diabetes duration, and smoking history. The results of the regression analyses are reported in terms of odds ratios (ORs), with corresponding 95% confidence intervals, and *p*‐value threshold for significance set at 5%.

## 4 RESULTS

Data on three hundred and forty patients (246 men and 94 women) were chosen for this study. Baseline characteristics are depicted in Table 1. The average patient age was 65 years. Of this group, 308 of patients (91%) had type 2 diabetes mellitus, while the remaining 32 (9%) had type 1 diabetes. Duration of diabetes was >10 years in 211 patients (71%), with 34 (12%) patients being diagnosed <5 years prior, and 52 (17%) being diagnosed 5–10 years ago. The most common comorbidities were hypertension (76%), hypercholesterolaemia (65%), and peripheral neuropathy (56%). 70% of patients lived with others, with 23% living alone, and 7% living in a nursing home. There were also patients with pre‐existing vascular disease, some of whom had previously had interventions performed prior to this admission. For this admission to the DFU, the location of the diabetic ulcer was divided between toes (59%) and foot/ankle/leg (41%). 30% of patients were diagnosed with osteomyelitis, and 8% had bilateral foot ulcers.

Patients were divided into two groups: those who received medical management and those who received surgical management. There was incomplete information on two patients (one male and one female), and they were not included in further analysis. Univariate logistical regression was then performed on the two groups, as shown in Table 2. Increased association with surgical management (OR >1) were found in four data points: HbA1c, CRP level, presence of osteomyelitis, and length of stay. One data point was found to clearly have a decreased association (OR <1): history of peripheral artery disease (PAD). Significance was shown with all these findings showing *p* < 0.05. No significant associations were found between groups in BMI, diabetes type and duration, presence of peripheral neuropathy, ulcer location, below knee arterial disease, smoking history, or history of open or endovascular revascularisation procedures.

**TABLE 2:** Univariate logistic regression: Medical versus surgical management.

	Medical (control)	Surgical (cases)	OR	95% CI	*p*‐value
	(*n* = 172)	(*n* = 166)			
Age (years)					
Median (IQR)	64.5 (30, 89)	65 (33, 86)	0.99	0.98–1.01	0.664
Gender (%)					
Female	43 (25%)	50 (30%)	0.77	0.48–1.25	0.292
Male	129 (75%)	116 (70%)			
Body mass index (kg/m^2^)					
Median (IQR)	29.5 (20.0, 45.7)	29.8 (19.7, 53.0)	1.01	0.98–1.04	0.436
C‐reactive protein (mg/L)					
Median (IQR)	20 (1.3, 180.0)	64 (1.7, 327.0)	1.01	1.00–1.02	<0.001
<5 (%)	24 (14%)	15 (9%)	1.66	0.83–3.28	0.144
>5 (%)	145 (86%)	150 (91%)			
Length of stay (days)					
Median (IQR)	7 (0.0, 28.0)	11 (4.0, 58.0)	1.08	1.05–1.12	<0.001
Diabetes status					
DM type (%)					
1	19 (11%)	13 (8%)	1.46	0.70–3.06	0.311
2	153 (89%)	153 (92%)			
DM duration (years) (%)					
<5	16 (11%)	18 (12%)	1.13	0.57–2.21	0.732
1–10	23 (15%)	29 (20%)	1.12	0.47–2.67	0.797
>10	112 (74%)	98 (68%)	0.77	0.38–1.61	0.497
HbA1c %					
Median (IQR)	8.0 (5.5, 12.7)	8.3 (5.4, 14.6)	1.11	1.00–1.22	0.042
Living situation					
Lives alone	35 (21%)	42 (26%)	1.2	0.77–1.88	0.426
Lives with others	124 (73%)	109 (67%)	0.73	0.44–1.23	0.238
Nursing home	11 (6%)	12 (7%)	0.91	0.36–2.31	0.841
Co‐morbidities					
Hypertension (%)	126 (74%)	128 (77%)	1.18	0.71–1.94	0.523
Hypercholesterolaemia (%)	119 (70%)	100 (60%)	0.66	0.422–1.04	0.072
Peripheral neuropathy (%)	92 (54%)	97 (59%)	1.22	0.80–1.89	0.357
Peripheral arterial disease (%)	73 (49%)	50 (35%)	0.56	0.35–0.91	0.018
Ischaemic heart disease (%)	64 (38%)	57 (34%)	0.84	0.54–1.32	0.458
Cerebrovascular accident (%)	37 (22%)	27 (16%)	0.70	0.40–1.21	0.199
Smoking					
Never smoker	75 (45%)	68 (42%)	0.91	0.65–1.26	0.558
Ex‐smoker	62 (37%)	72 (45%)	1.28	0.80–2.05	0.304
Current smoker	30 (18%)	21 (13%)	0.77	0.40–1.47	0.433
Previous vascular interventions					
Open revascularisation (%)	12 (7%)	7 (4%)	0.55	0.21–1.44	0.214
Endovascular procedure (%)	44 (26%)	31 (19%)	0.65	0.38–1.09	0.099
Treated iliac disease (%)	8 (5%)	1 (1%)	0.12	0.02–1.00	0.136
Treated above knee disease (%)	22 (13%)	25 (15%)	1.22	0.66–2.27	0.517
Treated below knee disease (%)	29 (18%)	24 (15%)	0.82	0.45–1.48	0.514
Ulcer characteristics					
Ulcer location (%)					
Forefoot	92 (55%)	99 (63%)	1.37	0.88–2.14	0.163
Rest of foot/ankle/leg	74 (45%)	58 (37%)			
Osteomyelitis (%)	38 (23%)	62 (38%)	2.12	1.32–3.43	0.002
Bilateral foot ulcers (%)	16 (9%)	10 (6%)	0.63	0.28–1.42	0.256
Vascular status					
Aorto‐iliac disease (%)	10 (9%)	6 (7%)	0.77	0.27–2.22	0.631
Above knee arterial disease (%)	50 (47%)	46 (55%)	1.42	0.80–2.52	0.234
Below knee arterial disease (%)	75 (46%)	57 (36%)	0.66	0.42–1.03	0.066

Abbreviations: DM, Diabetes Mellitus; IQR, interquartile range.

The median HbA1c level in the medical group was 8.05% compared to the surgical group of 8.3%. This difference was also found to be significant with OR 1.11, *p* = 0.042. The median CRP in patients who received medical management was 20, while for patients who received surgical management it was 64, which resulted in an OR 1.01, *p*‐value <0.001. Osteomyelitis at the ulcer site also notably increased the likelihood of surgery (OR 2.12, *p* = 0.002). Overall median inpatient length of stay was 5 days (IQR 0–58). The median length of stay for the medical group was 7 days (IQR 0–28), with the surgical patients staying for a median length of 11 days (IQR 4–58). This was found to be significantly different with OR 1.08, *p*‐value <0.001. Results showed that patients with a known history of PAD were more likely to receive medical management, with 49% of patients in the medical management group reporting a history of PAD; in comparison, 35% of patients receiving surgical management had a history of PAD (OR 0.56, *p* = 0.018).

When multivariate analysis was carried out, three factors were found to be associated with surgical intervention (Table 3). An elevated CRP was found to be associated with surgical management (OR 1.01, 95% CI: 1.01–1.02, *p* < 0.001), as was ulceration at the forefoot compared to elsewhere on the foot or leg (OR = 2.19, 95% CI: 1.13–4.23, and *p* = 0.019). Patients with Osteomyelitis (OM) were shown to have the strongest association with surgical intervention (OR 2.22, 95% CI: 1.14–4.34, and *p* = 0.019). This analysis was adjusted for age, gender, diabetes type, diabetes duration, and smoking history.

**TABLE 3:** Multivariate logistic regression: Medical versus surgical management.

Variable	OR	95% CI	*p*‐value
C‐reactive protein (mg/L)	1.01	1.01–1.02	<0.001^a^
Ulcer location (forefoot)	2.19	1.13–4.23	0.019
Osteomyelitis	2.22	1.14–4.34	0.019

*Note*: Adjusted for age, gender, diabetes type, diabetes duration, and smoking history.

^a^ Bonferroni adjustment of significance <0.002.

## 5 DISCUSSION

This study demonstrates several significant associations for patients with DFD undergoing either medical or surgical management. Knowing these associations may assist in minimising unnecessary intervention and reducing hospital length of stay, as well as improving patient care. Factors associated with surgical management in univariate analysis were HbA1c, CRP level, presence of osteomyelitis in diabetic foot ulcers, and length of stay. Of these factors, CRP level and the presence of OM and forefoot ulcers were also found to be associated with surgical management in multivariate analysis.

Apart from the patients who require lifesaving emergent surgery, the optimal management of less severe DFD complications is of ongoing controversy. Several reviews have been published without a definitive conclusion [6, 7, 8] on the efficacy and preference of one strategy over another due to the lack of direct comparison and randomised controlled trials. Some clinicians advocate for a predominantly surgical approach and do not regard conservative medical management by antibiotic therapy as ‘curative’, with poorer outcomes, longer healing time and reduced overall healing with higher risks of major amputation [9, 10]. Others have found no difference in healing rates, time to healing, and short‐term complications for patients who received antibiotics alone [11, 12].

Zeun et al. [11] reported that 64% of patients achieved complete healing and remission of OM at the end of the 12 months through medical management. Similar healing rates were observed in the study by Acharya et al. [12]. An expert review of anti‐infective therapy has found that antibiotic therapy appears to be as safe and effective as surgery at managing uncomplicated forefoot OM [6]. One randomised comparative trial reported that antibiotic therapy and surgical treatment has similar outcomes in patients with neuropathic forefoot ulcers complicated by OM [13]. However, this study had significant exclusion criteria which included necrotising soft tissue infections, accompanying OM, PAD, Charcot foot, Hba1c >10%, bone exposure within the ulcer, pregnancy, renal and hepatic impairments, and antibiotic allergies. However, the authors of this paper feel that such studies are not reflective of the many patients that present with DFD. As such, further insight into factors that can improve clinical decision making may be of benefit, such as those that have been shown in this study.

Univariate analysis demonstrated that patients with a raised HbA1c are more likely to require surgical management. While marginal, this can be interpreted as an increased risk for patients with more poorly controlled diabetes, this is due to poor adherence or other factors influencing management. This in turn may cause patients to be at higher risk for more severe infection that requires surgical management. However, HbA1c was not found to be a significant predictor of surgical management in multivariate analysis, so this interpretation may be considered less clear, or its true effect masked by the presence of more significant comorbidities such as osteomyelitis and elevated CRPs.

Length of stay was also increased for patients who underwent surgical management. Of the three hundred and forty patients, approximately half (166) required surgical management with debridement or amputation. Data demonstrate that surgical patients stayed 4 days longer with a median stay of 11 days compared to 7 days for medical patients. Considering these patients underwent more interventions, this is an understandable result, and it also suggests that optimising medical management and avoiding surgical management would reduce patient length of stay. As such, early selection of those patients who would eventually require surgical debridement has the potential of reducing morbidity, length of stay, and associated cost.

Statistical analysis demonstrated several associations between patient factors and surgical management, but only one factor that was more strongly associated with medical management. This was for patients with a previous diagnosis of peripheral arterial disease. It can be inferred that, with this diagnosis, patients are more likely to be linked in with community and outpatient services to manage their condition. As such, they may be able to be present earlier, and with less severe infections, than others without this support.

It is important to note that the potential confounding effect of previous vascular interventions in the DFD population. In this clinical setting, regarding patients with DFD, it is not unreasonable to think that these patients will need repeat intervention(s) of their vasculature to heal their wounds. However, this does not appear to be the case in this instance. Statistically there was no difference between surgical and conservatively managed groups in our study. It is important to note that this may be different in a larger study or in a different population group; and this would be worth investigating in another study.

Multivariate analysis demonstrated that patients who underwent surgery were more likely to have an elevated CRP than those who underwent medical management. Statistically, this association is the least strong of those found in multivariate analysis (OR = 1.01), but it is statistically very significant (*p* < 0.001). This can be understood to make sense in the context of patients with more severe infection, and patients with OM, being more likely to require surgical management. Whilst the CRP level was gathered on admission, it is known that CRP does lag and may not be entirely representative of the degree of inflammation. As such, an area for future research may involve comparing peak CRP to the admission CRP to aid in predicting the need for operative intervention.

Presence of OM was shown to be the factor that was most strongly associated with surgical management in multivariate analysis (OR = 2.22), which is again in‐keeping with the understanding that patients with more severe DFD and more severe infection are more likely to require surgical management. Other, smaller studies that included less patients than this study, have also shown a positive association between elevated CRP on admission and amputation rates; however, neither of these studies included the presence of osteomyelitis [14, 15].

The third factor associated with surgical management (debridement or amputation), in multivariate analysis is ulcers in the forefoot location. This association was also strong (OR = 2.19, *p* < 0.019). The authors of this paper believe that timely surgery aims to control infection and minimise soft tissue spread, as amputation is often necessary to manage gangrene or septic arthritis. It should be noted that wound debridement and toe amputations in this area can be viewed as a more minor procedure compared to more invasive operations such as above or below knee amputations.

Compared to major operations such as above or below knee amputations, wound debridement, and toe amputations (which are considered minor amputations) aim to preserve the major foot structures to allow biomechanical stability while removing dead tissue. This was demonstrated by Peters et al. [16], who found that patients with toe or midfoot amputations had similar functional impairment scores as patients without amputations. In contrast, functional impairment scores of patients with major lower limb amputations were significantly higher. A study focusing on health‐related quality of life [17] also found no difference between patients treated conservatively compared to those with minor amputations. The benefit of early intervention was studied by Tan et al. [5]. They found that patients who had prompt surgery, that is, surgical debridement or minor amputations, within 3 days of admission to hospital were less likely to require an above ankle amputation during the same hospitalisation period or within a follow‐up period of 1 year. As such, minor amputations (and wound debridement) may be considered as a viable treatment option for complicated DFD. Prompt identification and surgery in this group would reduce morbidities, length of stay and associated cost with minimal impact on lower limb functionality.

This study has the strength of a moderately large sample size collected over 2 years with a comprehensive data set. As the multidisciplinary DFU was the provider of specialised hospital‐based care, the risk of selection bias for either treatment group was reduced. However, the study had several limitations. Variables that could have affect outcomes include incomplete documentation of wound and infection severity, missing positive probe to bone tests and tissue microbiology. Socio‐economic status and patient frailty were also poorly documented making it difficult to accurately assess the impact of social factors as possible predictors of operative intervention. Data regarding theatre access and bed delays and cancellations which may have affected length of stay in surgical patients were also not recorded. The initial data was collected prior to standardised documentation and procedural protocol within the DFU; and provides an overview of a department in which there may be variations in decision making practices between clinicians.

Recent scoring systems such as the Wound, Ischaemia, and foot Infection (WIfI) classification [18, 19] is a validated tool predicting 1 year risk of major amputation in critical limb ischaemia and diabetic feet [18, 19]. Comprised of three clinical components–wound, ischaemia, and foot infection–limbs are categorised into one of four clinical stages with increasing risk of major amputation to provide prognostic stratification to guide decision making. The data of this study incorporate much of this, and at the time of the writing of this study are now increasing in use between the various clinicians in the DFU. Together with the advent of new scoring systems, this research has helped Monash Health formulate local guidelines to assist clinicians in the prompt recognition of DFD that will best serve the local community.

Additional areas of research could include actual use of WIfI scores and comparison between metropolitan and regional patients—to incorporate the social determinants that affect regional patients (especially access to community services—such as regular podiatry, diabetic surveillance) as well as delays to accessing beds in a tertiary centre. Research could include further analysis in patients that have undergone open and, or endovascular interventions without amputation and whether such interventions prevent further amputations in patients with known OM, high CRP, and forefoot ulcers.

The Monash DFU treats patients from a large catchment area in south‐east Melbourne and the greater Gippsland region. Prevalence of diabetes in these regions is 1.8% higher than the national prevalence of 6% and has some of the most socio‐economically disadvantaged local government areas, affecting the rates of hospital separations with a greater percentage of males and higher rates of PAD and foot amputation [20]. As such, this may not be representative of the typical pattern of complicated DFD in Australia. This study would benefit from comparison with other regions and with complete documentation of the variables mentioned. While this study may help identify patients needing early surgical intervention, further research is required to corroborate the findings of this study in other populations as well as its long‐term benefits and its health‐economic impact. It should be noted that within the Monash Health catchment area there are a significant number of patients of Aboriginal or Torres Strait Islander (ATSI) background. This is a population group that is known to have higher rates and incidence of diabetes and diabetic complications. This study did not analyse patients on their race or ATSI background, leaving scope for further research and analysis that should enable future clinicians in minimising the health inequity that affects this population group.

## 6 CONCLUSION

This study has found that elevated CRP levels, ulcer location on the toe, and osteomyelitis are strongly associated with surgical interventions during hospital admission. Patients with these three factors would benefit from consideration of early patient‐centred interventions with the potential of reducing inpatient stay. Identification of these factors in patients presenting with complicated DFD early in the admission course followed by prompt surgical intervention may reduce morbidities and length of stay and prevent unnecessary delays to care. Furthermore, this study will help clinicians in centres that do not have a DFU to recognise such patients in order to enable expedient transfers to centres that are best equipped to look after them.

## AUTHOR CONTRIBUTIONS

**Amos Au:** Analysis, interpretation of data and writing of article. **Erwin Yii:** Analysis, interpretation of data, framework. **Ana Andric:** Conception and design, analysis, interpretation of data. **Jennifer Wong:** Conception and design, review. **Alan Saunder:** Conception and design, review. **Ming Yii:** Conception and design, review and drafting.

## CONFLICT OF INTEREST STATEMENT

No conflicts of interests, no monetary gains to declare.

## ETHICS STATEMENT

This was granted by the Monash Health Ethics committee.

## Data Availability

The data that support the findings of this study are available from the corresponding author upon reasonable request.
